# Modeling healthy male white matter and myelin development: 3 through 60 months of age^☆^

**DOI:** 10.1016/j.neuroimage.2013.09.058

**Published:** 2014-01-01

**Authors:** Douglas C. Dean, Jonathan O'Muircheartaigh, Holly Dirks, Nicole Waskiewicz, Katie Lehman, Lindsay Walker, Michelle Han, Sean C.L. Deoni

**Affiliations:** a Advanced Baby Imaging Lab, School of Engineering, Brown University, Providence, RI 02912, USA; b Department of Neuroimaging, King's College London, Institute of Psychiatry, De Crespigny Park, London SE5 8AF, UK

**Keywords:** Brain development, Infant imaging, Myelin maturation, Myelin water fraction, White matter development

## Abstract

An emerging hypothesis in developmental and behavioral disorders is that they arise from disorganized brain messaging or reduced connectivity. Given the importance of myelin to efficient brain communication, characterization of myelin development in infancy and childhood may provide salient information related to early connectivity deficits. In this work, we investigate regional and whole brain growth trajectories of the myelin water fraction, a quantitative magnetic resonance imaging measure sensitive and specific to myelin content, in data acquired from 122 healthy male children from 3 to 60 months of age. We examine common growth functions to find the most representative model of myelin maturation and subsequently use the best of these models to develop a continuous population-averaged, four-dimensional model of normative myelination. Through comparisons with an independent sample of 63 male children across the same age span, we show that the developed model is representative of this population. This work contributes to understanding the trajectory of myelination in healthy infants and toddlers, furthering our knowledge of early brain development, and provides a model that may be useful for identifying developmental abnormalities.

## Highlights

• Proposes various growth models for modeling MWF trajectories
• Demonstrates modeling of MWF trajectories of 122 male infants under 5 years
• Statistically compares tested models using Bayesian information criterion
• Generates first four-dimensional model of whole-brain myelination of early childhood
• Discusses the utility of developed model

## Introduction

The elaboration of the myelin sheath around neuronal axons, and the associated white matter maturation, is a cornerstone of human neurodevelopment. Myelinated white matter forms efficient communication pathways that shape the integrated neural systems responsible for higher order functioning (Fornari et al., 2007, Johnson and Munakata, 2005). Given myelin's critical role in brain communication, processes that disrupt its development may result in reduced brain connectivity and inefficient interneuronal communication. In turn, this may lead to altered neuronal functioning, and may contribute to some neurodevelopmental and psychiatric disorders, including autism and attention deficit and hyperactivity disorder (Courchesne, 2004, Haroutunian and Davis, 2007, Konrad and Eickhoff, 2010).

Myelination during the first five years of life is a rapid and dynamic process. Prior histological studies have established that myelination begins in the cerebellum and brainstem in utero (Barkovich et al., 1988, Flechsig, 1901, Paus et al., 2001, Yakovlev and Lecours, 1967). Following birth, myelination proceeds caudocranially from the splenium of the corpus callosum, optic radiations and internal capsule by 3–4 months; occipital and parietal lobes by 5–6 months; temporal and frontal lobes by 9–11 months (Flechsig, 1901, Yakovlev and Lecours, 1967); and continues into the second decade of life (Barnea-Goraly et al., 2005, Bartzokis et al., 2010). However, while retrospective histological studies provide the most faithful characterization of myelin development, they suffer significant limitations. They are i) inherently cross-sectional; ii) difficult to combine into a single temporal timeline, owing to differences in staining techniques and inconsistent brain coverage; iii) difficult to obtain from large specimen pools spanning the infant age-range; iv) preclude investigation of underlying structure–function relationships; and v) labor intensive. Further, they may not necessarily reflect healthy development as these studies are conducted post-mortem.

Recent in vivo magnetic resonance imaging (MRI) techniques, including conventional T_1_- and T_2_-weighted structural imaging, diffusion tensor (DT)-MRI, and magnetization transfer imaging (MTI), have become popular for investigating early brain development (Giedd and Rapoport, 2010, Giedd et al., 1999, Knickmeyer et al., 2008) and, especially, white matter maturation (Geng et al., 2012, Lebel et al., 2012, van Buchem et al., 2001). These non-invasive techniques provide detailed anatomical tissue contrast and micro-structural insight that affords a more sensitive and direct means of examining white matter development. However, these methods also have their disadvantages. While conventional MRI (T_1_- and T_2_-weighting) have shown alterations in the gray/white matter contrast (Huang et al., 2006, Paus et al., 2001) temporally mirroring myelination, these qualitative observations are influenced by a variety of micro-structural and biochemical elements (MacKay et al., 2009). DT-MRI offers a quantitative approach, with metrics including fractional anisotropy (FA), mean diffusivity (MD), and axonal and radial diffusivity (AD and RD, respectively). Changes in these metrics during development have often been associated with myelination, however these measures are also associated with changes in the local tissue architecture (Jones et al., 2013, Mädler et al., 2008). Many of these measures are also derived directly from the tensor model of diffusion that does not apply to all brain voxels, making the interpretation difficult (Wheeler-Kingshott and Cercignani, 2009). Similarly, while the magnetization transfer ratio (MTR) has been shown to correlate strongly with myelin content (Moll et al., 2011, van Buchem et al., 2001, Zaaraoui et al., 2008), the MTR is also influenced by other processes including edema and/or inflammation (Gareau et al., 2000, Vavasour et al., 2011).

Multi-component analysis of relaxation time data, also termed multi-component relaxometry (MCR), may provide a more sensitive measure of myelin content. MCR decomposes the measured MRI signal into contributions from distinct micro-structural water compartments. Prior MCR studies have consistently reported at least two water compartments: a fast-relaxing water pool attributed to water trapped between the myelin-lipid bilayers; and a slower-relaxing water pool attributed to intra-/extra-cellular water (MacKay et al., 2006, Whittall et al., 1997). Quantification of the signal from the myelin-bound water, termed the myelin water volume fraction (MWF), has been shown to strongly correlate with histological assessments of myelin content (Gareau et al., 2000, Laule et al., 2006, Laule et al., 2008, Odrobina et al., 2005, Webb et al., 2003) and provide improved myelin specificity compared to DT-MRI measures or MTR (Gareau et al., 2000, Mädler et al., 2008, Vavasour et al., 2005).

While MCR has traditionally been performed using multi-echo T2 decay data, a more recent approach, termed mcDESPOT (multi-component driven equilibrium single pulse observation of T_1_ and T_2_), has been proposed (Deoni et al., 2008). Though at the expense of a more complicated signal model that must include the effects of water exchange, mcDESPOT offers the potential advantages of improved SNR, reduced acquisition times, and increased spatial resolution and volumetric coverage compared to the established T_2_ approach. While comparison of multi-echo T_2_ and mcDESPOT MWF values present a known discrepancy, with mcDESPOT values being consistently larger (Zhang et al., 2013), they do, however, correspond qualitatively with histological myelin content measures in a Shaking Pup model of dysmyelination (Hurley et al., 2010), and have been used to investigate structure–function impairment in MS (Kitzler et al., 2012, Kolind et al., 2012) and other demyelinating disorders (Kolind et al., 2013). More recently, the mcDESPOT has been applied to the study of white matter maturation and healthy infant neurodevelopment (Deoni et al., 2011, Deoni et al., 2012), revealing a strong consistency with the known spatial–temporal pattern of myelination.

A continuous and probabilistic model of myelination could alleviate these concerns. Derivation of an appropriate growth model would allow estimation of the typical mean MWF, and variance, for any age. This work sought to develop such a model by comparing common growth functions fit to measured MWF data. The most appropriate model was then used to generate a continuous, four-dimensional “atlas” of healthy MWF development, allowing calculation of the ‘typical’ average and standard deviation MWF maps at any desired age. As proof-of-concept, individual and group-averaged MWF maps were statistically compared to the growth model derived MWF maps, with no significant differences found. The developed atlas, therefore, represents the first continuous model of myelin maturation in healthy male infants; provides an important step for understanding the typical myelination trajectory; and provides a framework from which to identify the earliest of white matter changes.

## Materials and methods

### Subjects

MRI data analyzed in this work are part of an ongoing longitudinal study investigating white matter maturation in healthy, typically developing children and its relationship to behavioral development (Deoni et al., 2012). Informed parental consent was obtained in accordance to ethics approval from the Institutional Review Board of the host institution. Enrolled infants met the following inclusion criteria: uncomplicated single birth between 37 and 42 weeks; no exposure to alcohol or illicit drugs during pregnancy; no familial history of major psychiatric or depressive illness; no diagnosis of major psychiatric, depressive or learning disorder in participant; and no pre-existing neurological conditions or major head trauma. In total, 122 healthy male infants and toddlers between 70 and 1809 days of age (mean = 690.14 days, corrected for a 40-week gestation) were analyzed. Table 1 provides an age-group break down of these participants.

**Table 1:** Age information of the 122 scanned participants. Ages given are given in days and corrected for gestational period.

	3 months	6 months	9 months	12 months	15 months	18 months	21 months	24 months	30 months	36 months	42 months	48 months	54 months	60 months
Number of subjects	21	15	11	6	5	5	6	2	15	9	7	9	6	5
Age (range)	70–124	140–211	240–313	316–383	413–487	497–573	593–677	720–724	772–987	1030–1728	1205–1328	1368–1526	1541–1683	1728–1809
Age (mean)	101.67	178.53	271.91	346.17	466.4	522.6	634	722.00	887.87	1158.56	1284.71	1430.22	1620.67	1758.00
Gestational period (mean)	39.31	39.58	39.51	39.95	39.8	39.54	40.17	37.14	39.47	39.02	39.84	38.90	39.29	39.51

### Measuring MWF in infants

Whole-brain MWF maps were acquired using the rapid mcDESPOT (Deoni et al., 2008) imaging technique. Imaging protocols for this age-range, using acoustically-muffled sequences, have been presented previously (Deoni et al., 2011, Deoni et al., 2012), and comprise 8 T_1_-weighted spoiled gradient echo images (SPGR or spoiled FLASH), 2 inversion-prepared (IR)-SPGR images and 16 T_1_/T_2_-weighted steady-state free precession (SSFP or TrueFISP) images.

MWF maps were calculated from these data using a three-pool signal model estimating intra/extra-axonal water; myelin-associated water; and a non-exchanging free water pool (Deoni et al., 2013). Corrections for flip angle (B_1_) and off-resonance (B_0_) inhomogeneities were also performed (Deoni, 2010). Total imaging times ranged from 19 min for the youngest infants to 24 min for older and larger children.

Children under 4 years of age were scanned during natural, non-sedated, sleep; while children over this age were able to watch a favorite TV show or movie. All data was acquired on a 3 T Siemens Tim Trio scanner equipped with a 12 channel head RF array. To minimize intra-scan motion, children were swaddled with an appropriately sized pediatric MedVac vacuum immobilization bag (CFI Medical Solutions, USA) and foam cushions were placed around their head. Scanner noise was reduced by limiting the peak gradient amplitudes and slew-rates to 25 mT/m/s. A noise-insulating insert (Quiet Barrier HD Composite, UltraBarrier, USA) was also fitted to the inside of the scanner bore. MiniMuff pediatric ear covers and electrodynamic headphones (MR Confon, Germany) were used for all scanned children, while a pediatric pulse-oximetry system and infrared camera were used to continuously monitor the sleeping infants during scanning. After acquisition, image data was assessed for motion artifacts (blurring, ghosting, etc).

### MR analysis and myelin trajectory modeling

Following calculation of the 122 MWF maps, all maps were non-linearly aligned to a study specific template (Deoni et al., 2012) using the Advanced Normalization Tools software package (Avants et al., 2008) and smoothed with a 3 mm Gaussian kernel. Non-brain parenchyma was removed using FSL's brain extraction tool (BET) (Smith, 2002). Regional masks for bilateral frontal, temporal, parietal, and occipital lobes, cingulum, and cerebellar white matter, as well as the genu, splenium and body of the corpus callosum were derived from the MNI adult template (Mazziotta et al., 2001), co-registered to the study template, and superimposed upon each infant's MWF maps (Deoni et al., 2012). Mean MWF values for each mask were obtained for each infant and plotted against the infant's gestational-corrected age. To examine whether the nonlinear transformation affected the quantitative values, mean MWF values were also extracted from native space after applying the inverse warp transformation to the regional masks. These native-space values were then compared to the standard space values.

**Fig. 1:**
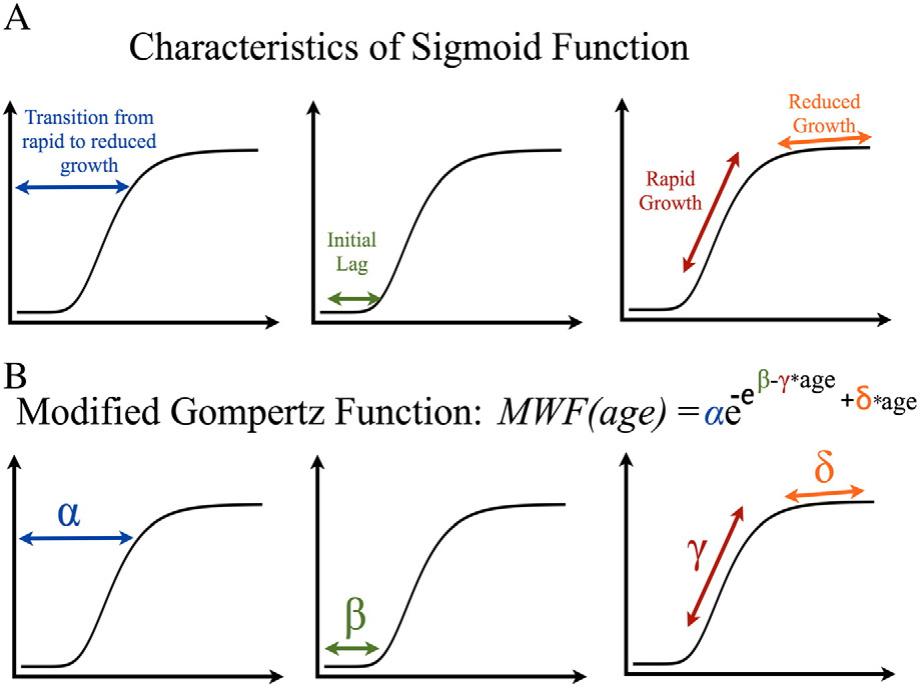
(A) Properties of a sigmoid function. Although many functional forms exist, each shares three similar characteristics: 1) an initial lag or period of slow growth, 2) a period of rapid exponential growth, and 3) a period of reduced growth-rate. These properties and shape of the overall curve are governed by the free parameters of the model. (B) *Modified* Gompertz model is described by 4 free parameters, each contributing to the characteristics of the curve. These parameters may be useful for describing biologically relevant metrics such as a developmental transitionary period (α), developmental lag, or (β) growth rates (γ, δ).

To the plotted data, the growth models of Gompertz (1825), Stannard et al. (1985), Richards (1959), Bleasdale and Nelder (1960), as well as more generic functions such as the logistic model, and hyperbolic tangent were fit. In addition to these, a modified form of the Gompertz model was also included in the group of growth models. Although diverse, each of these sigmoidal functions shares similar characteristics: 1) a lag or period of slow growth; 2) a period of rapid exponential growth; and 3) a period of reduced growth-rate (Fig. 1A). From these functions, biologically relevant metrics, such as lag period, growth rate, and maximum size, may be derived (Fig. 1A).

Non-linear regression via a Levenberg–Marquardt non-linear least squares (Levenberg, 1944) algorithm was used to determine the best fit for the free-parameters of each sigmoidal function (see Table 2). Fits of each model were compared using the Bayesian Information Criterion (BIC, Schwarz, 1978), a parsimony metric that compares the fit residuals while penalizing for the number of model parameters. The model that provided the lowest BIC measure consistently across the investigated brain regions was defined as the most representative.

**Table 2:** Fitting functions used to fit the myelination trajectories in children less than 2 years of age. The fitting routine first estimates the initial values for the free parameters and is then followed by non-linear regression that iteratively solves for the free parameter values by minimizing the sum of squares between the predicted and measured values. Free parameters are denoted by boldface Greek letters.

Functional form of modes		
Model	Number of parameters	Functional form of modes
Gompertz	3	**α** ∗ exp[− exp(**β** − **γ** ∗ x)]
Modified Gompertz	4	**α** ∗ exp[− exp(**β** − **γ** ∗ x) + **δ** ∗ x]
Bleasdale and Nelder	4	(**α** + **β**x**^δ^**)^− 1/**γ**^
Richards	4	**α** ∗ (1 + **β** ∗ exp[**γ** ∗ (**δ** − x)])^− 1/**δ**^
Stannard	4	**α** ∗ (1 + exp[−(**β** + **γ** ∗ x)/**δ**])^−**δ**^
Hyperbolic tangent	4	**α** + **β** ∗ tanh(**γ** ∗ x − **δ**)
Logistic	3	**α** ∗ [1 + exp(**β** − **γ** ∗ x)]^− 1^
General logistic	6	**α** + (**β** − **γ**) ∗ (1 + **ζ** ∗ exp[−**β** ∗ (x − **δ**)])^− 1/**ε**^

### Construction of a population-averaged MWF atlas

Using the BIC-selected ‘best’ model, voxel-wise fitting using wild-bootstrap with residual resampling (Efron, 1979) was performed to generate whole-brain, three-dimensional maps of the mean model parameters and their associated uncertainty. The resampling was performed 5000 times to provide accurate estimations of each parameter's distribution.

With this a priori knowledge of each parameter's mean, representative whole-brain MWF maps can be constructed for any age. Further, knowledge of the parameter uncertainty also allows calculation of the variance in this representative map as

(1) $$\mathrm{\delta MWF}\left(\mathrm{age}\right)=\sqrt{\sum_{i=0}^{i=N}{\left(\frac{\mathrm{\delta MWF}}{\delta a_{i}}\left|{}_{\mathrm{Age}}\right)\right)}^{2}}\delta a_{i}$$

where $\mathrm{\delta MWF}/\delta a_{i}$ represents the partial derivative of the growth model with respect to the *i*^*th*^ model parameter, $\delta a_{i}$ is the uncertainty of the *i*^*th*^ parameter, and N is the number of free parameters in the model. Individuals can be directly compared to this population-derived model, e.g., using Z-statistics, without necessitating age-grouping or requiring substantive study sizes.

### Comparison of MWF atlas to grouped and individually measured MWF data

To illustrate the similarity between model-derived and in vivo acquired MWF maps, two main analyses were performed on an independent sample of 63 male children. Subjects for these analyses were not included in the group of 122 participants used in generating the model and met the same inclusion/exclusion criteria as mentioned previously. Table 3 provides supplemental information on these additional subjects.

**Table 3:** Supplemental information on additional subjects used in comparing model-derived MWF maps to in vivo MWF maps. Comparison of model-derived MWF maps to group averaged MWF maps were broken into 9 separate age groups.

Model-derived MWF map comparison to group averaged MWF maps									
	Group 1	Group 2	Group 3	Group 4	Group 5	Group 6	Group 7	Group 8	Group 9
Number of subjects	4	8	9	7	5	6	6	4	10
Age (range)	106–141	253–309	312–396	410–477	502–583	593–652	712–942	1263–1294	1351–1809
Age (mean)	121.75	280.625	350.89	445.86	536.6	624.83	815.5	1278	1645.3


Model-derived MWF map comparison to individual MWF maps				
	Subject 1	Subject 2	Subject 3	Subject 4
Scan 1 age (days)	115	200	265	724
Scan 2 age (days)	288	353	449	1083
Scan 3 age (days)	449	578	632	1413

First, we examined the model's ability to examine average group differences. In vivo comparison data was broken into 9 different age groups (Table 3) and compared with age-matched model-derived mean MWF and *δMWF* maps. In vivo mean and standard deviation MWF maps were generated by calculating the mean and standard deviation of the MWF for each age group (Table 3). Paired t-tests were performed using FSL's Randomise tool (www.fmrib.ox.ac.uk/fsl/) to compare these in vivo and model-derived data, with significant differences defined as p < 0.05, uncorrected for multiple comparisons. Permutation testing was restricted to a custom white matter mask for each group. This custom white matter mask was created using the mean in vivo MWF map (for each group) by thresholding voxels with a MWF value below 0.02.

Next, the utility of using model-derived MWF maps to investigate individual differences was examined. The developed MWF model was compared to four individuals from which three repeated datasets have been acquired. Repeated measurements were acquired from these individuals as part of the longitudinal study protocol (Deoni et al., 2012). For each repeated measurement, age-matched model-derived MWF and maps were calculated. Individually measured MWF maps were normalized into the common study template space as previously described. Z-statistic analysis was then performed to identify differences between the acquired and model MWF maps. The Z-statistic was calculated at each imaging voxel:

(2) $$Z_{i}=\left(x_{i}-u_{i}/s_{i}\right)$$

where, *x*_*i*_ represents the *i*^*th*^ MWF voxel from the in vivo MWF map, *u*_*i*_ and *s*_*i*_ are corresponding voxels from the model-derived MWF and *δMWF* maps, respectively. Z-statistic analysis was also restricted to a white matter mask, created by thresholding voxels below with a MWF value below 0.02 from the individual's MWF map. Areas of significant deviation (p < 0.05, uncorrected for multiple comparisons) were defined as |*Z*| < 1.96.

### Post-hoc construction of confidence interval for corpus callosum

To further demonstrate that the selected model best characterizes the underlying growth trajectory of the MWF, 95% confidence intervals were constructed for the regional trajectories of the genu, body, and splenium of the corpus callosum. Non-linear fitting using the wild-bootstrap with residual resampling was performed on these three regions to generate mean and uncertainty measures for the parameters of the growth model from 5000 resamples. From these mean and uncertainty measures, 95% confidence intervals of the growth trajectory were constructed. Regional trajectories of the independent sample of 63 additional subjects previously mentioned (Table 3) were also compared to these normative model fits and confidence intervals. Residuals between the predicted (mean model) and measured (in vivo) MWF values were then calculated for each region.

## Results

**Fig. 2:**
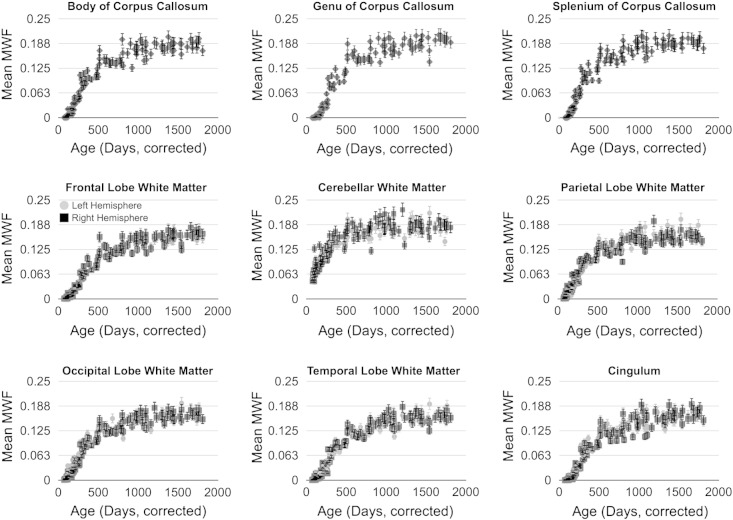
Derived myelination trajectories for the left and right hemisphere frontal, temporal, occipital, parietal and cerebellar white matter, as well as body, genu, and splenium of the corpus callosum from 122 healthy male infants under 5 years of age. Right and left hemisphere measurements are denoted with black squares and gray circles, respectively. Error bars represent the standard deviation of the measurement. These developmental trajectories exhibit the characteristic ‘S’-shaped curve of a sigmoid, suggesting this class of models might be best to characterize the underlying pattern.

**Fig. 3:**
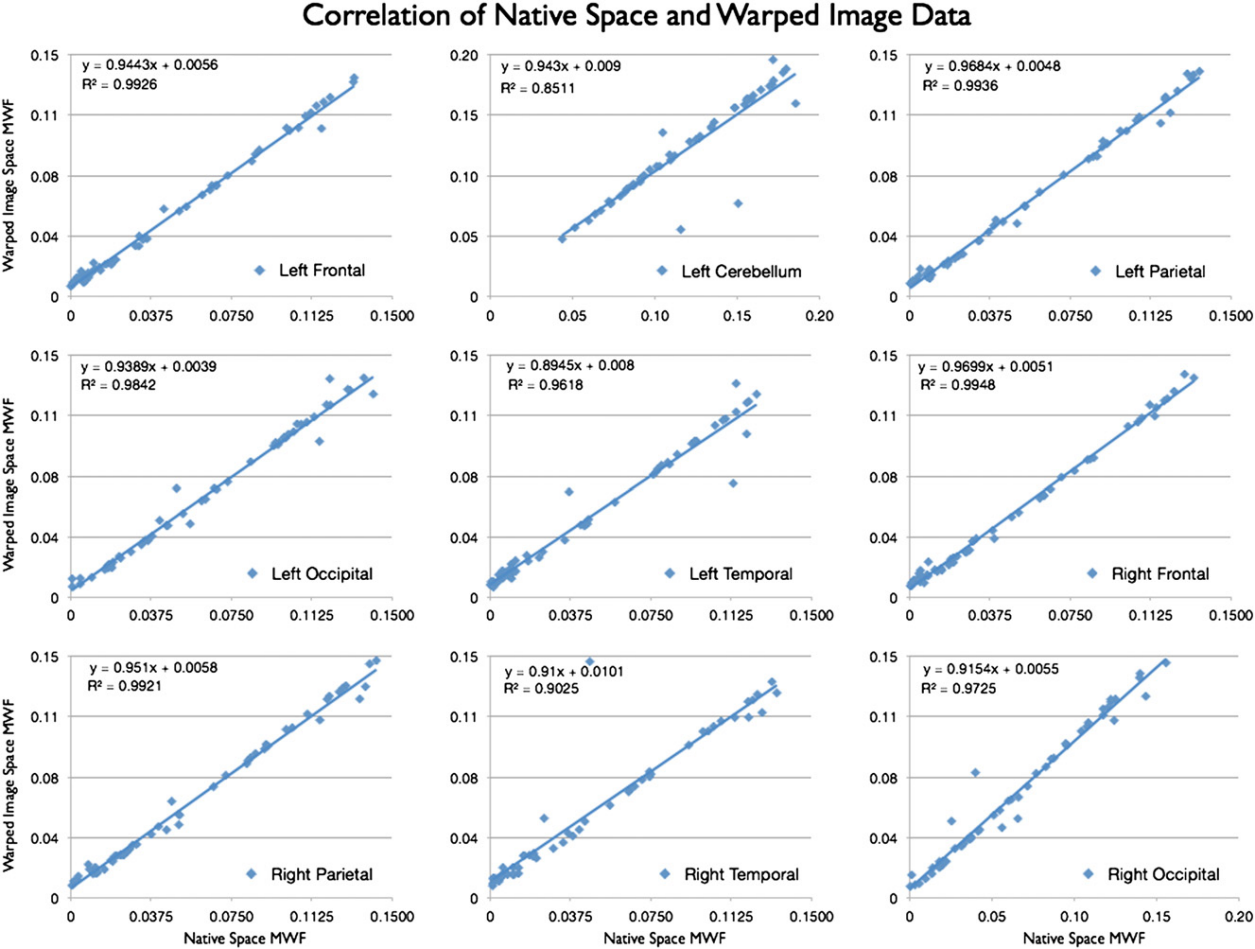
Plot comparing representative native-space and template-space mean MWF values. High correlation between the native-space and template-space illustrate that MWF values were not significantly altered through the normalization process.

Regional MWF trajectories for each investigated brain region are shown in Fig. 2. All trajectories follow the expected sigmoidal shape, with a period of lagged growth extending from birth through approx. 90–150 days, depending on the brain region, followed by rapid, exponential growth to approx. 400 days of age. Beyond 400 days, the trajectory begins to appear logarithmic in nature. We found transformed MWF values to be highly correlated with native-space values, with Pearson's r correlation coefficient ranging from 0.9225 to 0.9974 (Fig. 3). Thus, the normalization did not result in inhomogeneous changes to the MWF maps and consequently was found to be reliable for aligning individual MWF maps.

**Fig. 4:**
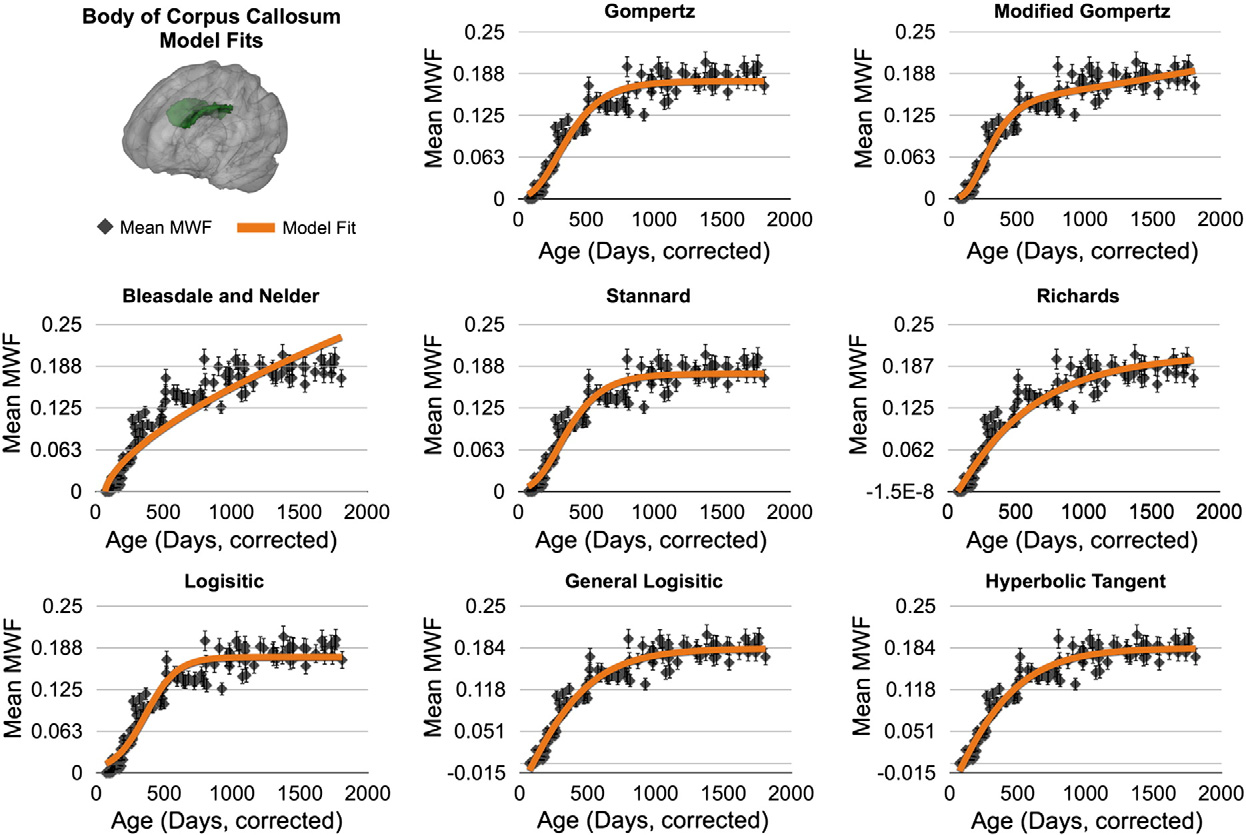
Representative fitting of the mean myelination trajectory for the body of corpus callosum. Blue points represent mean MWF values from the 122 male infants, while the red curve represents the best-fit curve for each investigated model. In total, 8 sigmoid models were examined. BIC metric values indicate that the *modified* Gompertz growth model provided the best fit.

A representative sample of the best-fit results for each model to data from the body of the corpus callosum is shown in Fig. 4. A summary of the entire fitting results, including free parameter estimates and BIC values, for each region is shown in Table 4. Based on BIC measure comparisons, we found that the modified Gompertz model consistently provided the most faithful characterization of the MWF data. Thus, this function was subsequently used to construct the 4-D developmental atlas by fitting this function to each individual voxel.

**Table 4:** BIC values for investigated ROIs and fitting functions. Values highlighted in bold typeface indicate the model that best fit the data.

Region	Gompertz	Modified Gompertz	Bleasdale & Nelder	Stannard	Richards	Logistic	General logistic	Hyperbolic tangent
Body of CC	61.027	**51.966**	130.787	68.032	68.879	81.048	63.046	53.977
Genu of CC	70.769	**55.949**	252.652	82.122	76.479	88.235	62.128	74.276
Splenium of CC	67.410	**54.001**	340.922	74.421	75.885	86.653	68.075	58.786
Right cerebellar WM	**49.022**	53.036	68.268	54.375	52.338	52.119	61.976	52.321
Left cerebellar WM	**65.687**	70.389	87.534	70.371	70.774	65.401	80.390	70.787
Right frontal WM	35.208	**28.558**	102.033	42.239	43.058	54.718	41.890	33.543
Left frontal WM	40.532	**29.578**	105.180	47.943	44.163	61.924	44.343	35.627
Right occipital WM	43.941	43.018	101.119	50.556	48.005	60.463	45.040	**35.595**
Left occipital WM	43.446	**42.028**	107.705	49.585	50.898	56.949	52.541	43.130
Right parietal WM	39.359	**36.017**	109.783	46.222	48.493	57.173	45.331	36.154
Left parietal WM	41.062	**34.827**	114.331	48.321	45.502	62.224	46.280	37.341
Right temporal WM	**35.452**	36.739	108.376	42.192	54.712	55.017	44.458	36.346
Left temporal WM	41.543	40.311	117.204	48.892	57.261	62.864	47.770	**39.517**
Right cingulum	65.705	**61.837**	99.781	73.378	64.982	84.783	68.782	57.379
Left cingulum	64.300	**48.183**	107.511	71.280	65.806	83.456	67.624	58.438

**Fig. 5:**
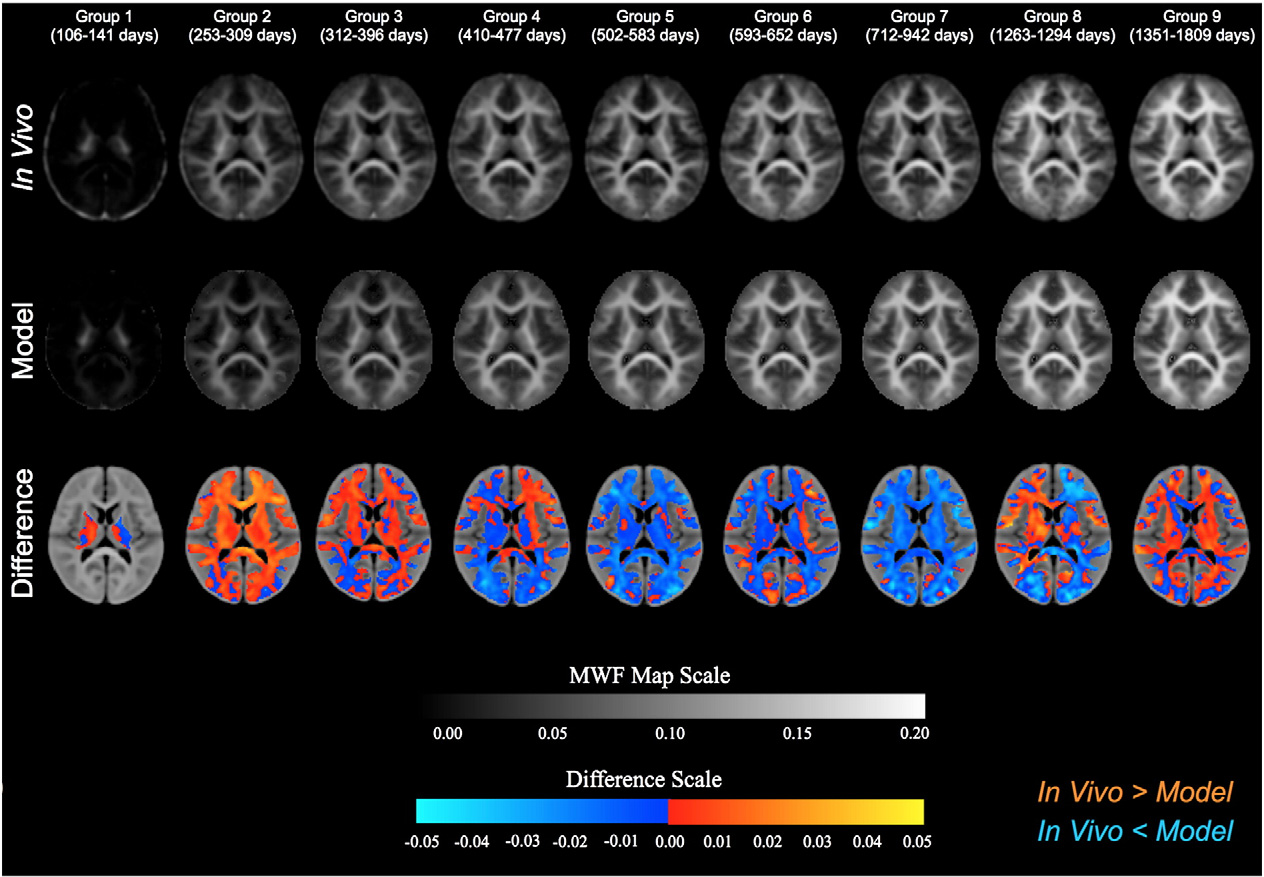
Mean, acquired, model-derived MWF maps, and difference maps for 9 separate age groups (Table 3). Comparison of acquired and model derived MWF maps was done using a paired *t*-test for each age group. Few significant differences (p < 0.05, uncorrected) were detected between the in vivo and model-derived maps.

**Fig. 6:**
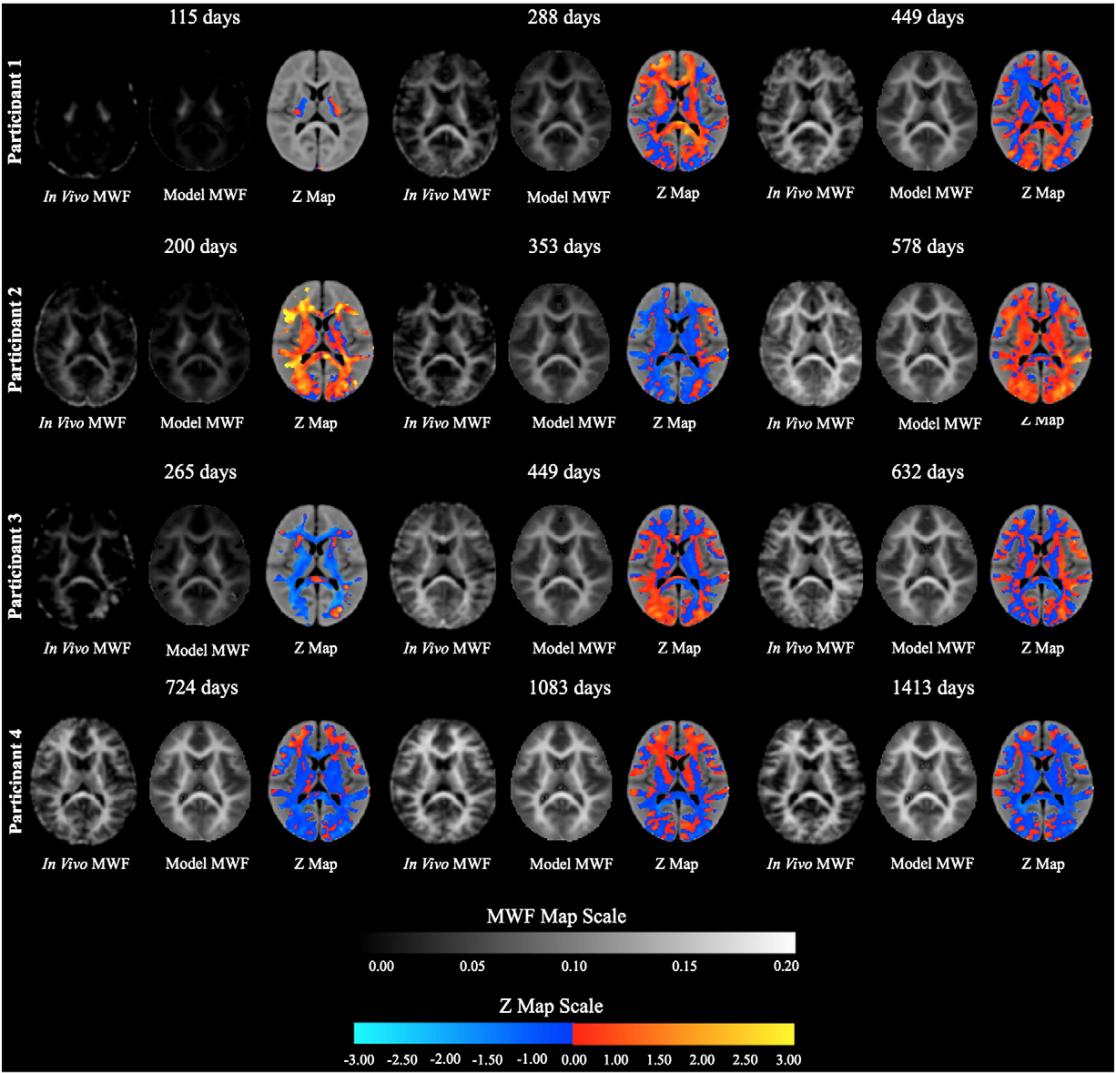
Repeated individual MWF maps at three different time points. Atlas derived MWF maps were derived for each corresponding age and compared to the longitudinal data using Z-statistics. Few areas of significant differences (|Z| < 1.96) between the repeated data and corresponding model-derived MWF maps were found.

The predictive validity of the model is shown in Fig. 5, Fig. 6. Fig. 5 contains results from the comparison of in vivo group-averaged MWF maps and model-derived maps at the same ages. Qualitatively, the in vivo and model-derived MWF maps appear similar. Results from the paired *t*-test analysis yielded few statistical differences (p < 0.05 uncorrected for multiple comparisons) between the acquired and model derived MWF maps for each age group.

We also investigated how well the atlas predicted individual MWF estimates at variable ages by comparing derived MWF maps with individually acquired MWF data. Representative results of these analyses are shown in Fig. 6, which shows images from the acquired maps, model-derived images, and the Z-statistic maps. Z-statistic values are indicated at each image voxel of the representative axial slice. A small number of regions of significant difference (|Z| < 1.96, corresponding to p < 0.05) were found between the acquired and model-derived MWF maps in the examined subjects.

**Fig. 7:**
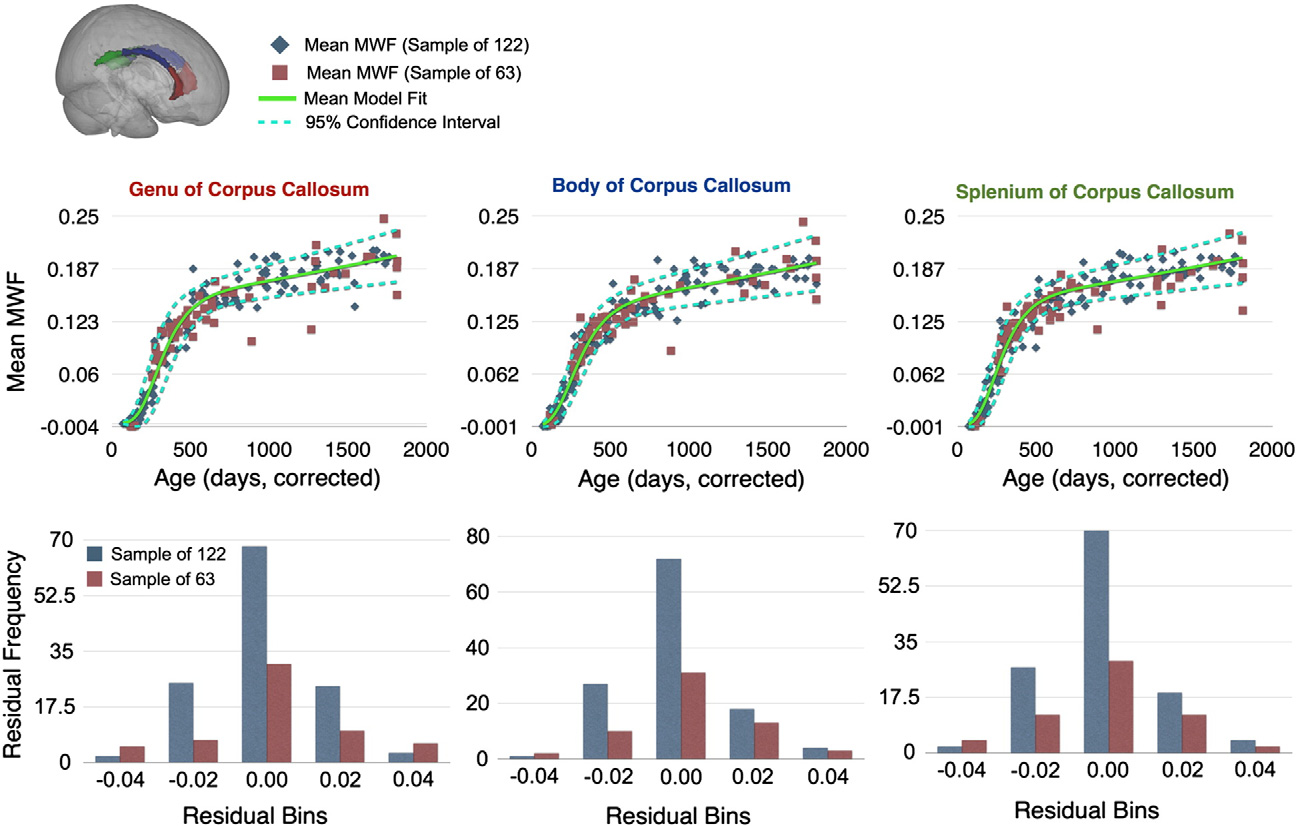
Bootstrapped mean Gompertz fit with 95% confidence interval for mean MWF values obtained from the genu, body and splenium of the corpus callosum.

Bootstrap analysis of the regional MWF trajectories for the genu, body and splenium of the corpus callosum was performed to construct 95% confidence intervals using the modified Gompertz model. The mean modified Gompertz fit with the 95% confidence intervals are shown overlaid on the trajectories of the three regions in Fig. 7. Residual histograms (Fig. 7) illustrate the distribution of the residuals across the trajectories of these three regions. As expected, bootstrapping the parametric fit of these regional trajectories resulted in approximately 95% of the individual measurements to be contained within the bounds of the confidence intervals and the residuals normally distributed (Fig. 7).

## Discussion

This work sought to model white matter myelination throughout the brain across the first five years of life in healthy male infants and toddlers. Investigating the overall shape of the myelination trajectories in the investigated regions revealed a non-linear sigmoidal developmental trajectory: with a lag period (0–150 days) preceding exponential growth (150–400 days) followed by a period of reduced growth thereafter. This growth trajectory is consistent with previous studies investigating this age-range (Deoni et al., 2012, Deoni et al., 2013, Hermoye et al., 2006). Of the sigmoidal models investigated, we found a modified version of the Gompertz function to provide the best characterization of the regional MWF developmental data. This result is also consistent with a prior study that used the traditional, 3 parameter Gompertz function to model DT-MRI metrics across a younger age-range (Sadeghi et al., 2012). However, this study did not investigate alternative functions, used data from only 3 age points and focused on a more restricted age range (under 2 years of age). Furthermore, while the traditional Gompertz function may be best to describe the underlying growth trajectory at an early age, this function approaches an asymptote as age increases, and thus limiting its ability to account for the continued myelination that occurs throughout childhood and early adulthood (Kumar et al., 2011, Lebel and Beaulieu, 2011). The inclusion of an additional linear term inside the exponent of the Gompertz function corrects for the asymptotic behavior of the Gompertz function, allowing the model to account for continued growth.

The biological interpretation of the modified Gompertz function's free parameters may provide important insight into the developmental process. Fig. 1B illustrates the role that each parameter has on the overall shape of this function. The first parameter, α, sets the overall scale of the function's trajectory and corresponds to the amplitude of the model at the transition from rapid, exponential development to reduced, continued growth. In terms of the current model, this parameter corresponds to the MWF value at which the developmental trajectory transitions from rapid, exponential growth to slow steady growth. The second parameter, β, describes the initial lag period of the model that corresponds to the time before rapid myelination. The third parameter, γ, describes the growth rate of the rapid exponential developmental period; while the fourth parameter, δ, corresponds to the growth rate of the slower, continued myelination observed to take place after 24 months of age. These measures are descriptive of the underlying maturational process and may be useful for future investigations of intrinsic growth patterns of white matter microstructure. For example, investigating the growth rate among brain regions would simply entail comparing the parameter γ of the interested regions. Furthermore, the age at which the MWF trajectory transitions from rapid, exponential growth to slow, continued development (characterized by α) may represent a pivotal time of the neurodevelopmental process (i.e. transition from infancy to toddler).

The development of a parametric growth model of myelination is also important for understanding linkages between evolving neurodevelopment and malbehavior, as well as clinical-relevance in premature infants, infants diagnosed with multiple sclerosis, among other complications. For example, children with autism have been reported to exhibit widespread atypical patterns of early brain growth, including accelerated maturation of white matter (Ben Bashat et al., 2007). MWF growth trajectories of children with autism could be directly compared to trajectories generated by the developed model in order to see where, when and how the growth trajectory deviates from the normative population. While additional work is required to evaluate comparisons with such populations, the robustness of the method demonstrated here strongly intimates its feasibility.

Although model MWF maps may be reconstructed after performing a single whole brain fit of the in vivo MWF data, the resulting fitting parameters contain no information regarding their uncertainty. To obtain information about the fitting parameter distribution, we utilized wild bootstrap resampling of the residuals. Bootstrap resampling is not new to MR imaging, as it has been used to estimate the uncertainty of fiber orientations from DT-MRI (Haroon et al., 2009, Jones, 2008, Yuan et al., 2008) and uncertainty of neural brain activity from functional MRI (fMRI) (Darki and Oghabian, 2009, Kirson et al., 2008). This non-parametric, model-based resampling method is advantageous because it does not require redundant data (i.e. multiple image acquisitions) in order to estimate standard errors and is sufficient to use in cases of homoscedasticity (uniform variance among data) and heteroscedasticity (non-uniform variance among data) (Chung et al., 2006). One potential caveat to this approach is that the model of choice (in this context, the modified Gompertz growth model) needs to appropriately characterize the measured data. However, our initial analysis of comparing a wide variety of growth functions, using BIC parsimony measures, examined this issue. We consistently found the modified Gompertz growth model to provide a better representation of the regional MWF developmental trajectories and therefore concluded that this model best represents the underling developmental pattern.

MWF imaging has a long history in the field of known demyelinating disorders, such as multiple sclerosis (MacKay et al., 2009), however, its use in examining structural and functional development is new. Further, mcDESPOT differs from the conventional and established techniques that have been verified histologically (Laule et al., 2006, Webb et al., 2003). Similar verification of mcDESPOT has been limited to histological comparisons in the Shaking Pup model of dysmyelination (Hurley et al., 2010) and indirectly through comparison with the known histological time-course of myelination in human infants (Deoni et al., 2011, Deoni et al., 2012), and demyelination studies in MS (Kitzler et al., 2012, Kolind et al., 2012). Thus, the specificity of mcDESPOT MWF measures as a reflection solely of myelin may be questioned. However, animal and in-vivo results garnered so far give confidence that if not specific to myelin, mcDESPOT provides novel information regarding white matter microstructure, and offers enhanced sensitivity to myelin changes relative to T_1_ and T_2_ relaxation times, or other measures.

Outliers, caused by intra-scan motion and other confounds, have the potential of influencing the calculation of the quantitative imaging maps (i.e. MWF maps) and therefore affecting the sum of squares fitting, which could lead to erroneous results (Chang et al., 2005). Further, these outliers may unreasonably inflate variability when performing wild bootstrapping. Motion artifacts were minimized as the scanning was performed during natural, non-sedated sleep. Images were further assessed for motion artifacts and none were found to be present. Other potential imaging-related confounds, such as B_0_ and B_1_ inhomogeneity, are accounted for within the mcDESPOT processing pipeline by mapping the B_1_ field and removing off-resonance effects by phase-cycling the bSSFP data (Deoni, 2010). A 3 mm full-width-at-half-maximum 3D Gaussian kernel was additionally used to spatially smooth the MWF maps of each participant. Although spatially smoothing the data reduces its effective spatial resolution, the kernel size chosen was reasonably conservative relative to comparable MRI studies, preserving image detail and minimally impacting MWF values. Qualitative inspection of the regional trajectories in Fig. 2 as well as the 95% confidence intervals and residual distributions constructed for the genu, body and splenium of the corpus callosum (Fig. 7) suggests that individual MWF measurements are well contained. Nonetheless, smoothing filter size has been shown to augment statistical neuroimaging findings (Jones et al., 2005) and therefore this parameter has an impact on the findings.

Quantifying the uncertainty of each parameter represents a critical step in the development of a model that describes MWF maturation. Mean and standard deviation estimates of each parameter can then be used to compute representative average and standard deviation MWF maps, for a given age between 70 and 1809 days, which can then be used to statistically compare against other typically developing and at-risk children in this age range. This aspect of the developed model was investigated using longitudinal measurements acquired in four subjects. The variation of the Z-statistic values observed in Fig. 6 suggests that there is individual deviation along the overall developmental trajectory. However, while this individual variation is known to exist (Giedd and Rapoport, 2010, Zilles and Amunts, 2013) and is therefore expected to be observed, a small number of these inter-individual deviations were found to be significant (|Z| < 1.96). In particular, examination of the first scan of subject 2 (Fig. 6) reveals areas of higher MWF in right hemispheric frontal and bilateral temporal white matter within the in vivo MWF map than the model-derived map. These results suggest that, at this point in time, the individual deviates from the normal myelination trajectory in these regions. The individual Z-statistic results suggest the developed MWF model to be representative of myelin maturation and sensitive to individual variation along the typical myelination trajectory. Moreover, although the paired *t*-test analysis of group averaged in vivo and model-derived MWF maps yielded few significant differences (p < 0.05, uncorrected), these differences did not remain significant after correcting for multiple comparisons using a cluster correction technique (FSL, cluster threshold of 1.96) in a post-hoc examination of these statistical results, suggesting the model-derived MWF maps to be representative of this normative population. This represents a significant new direction for investigating early neurodevelopment. Importantly it provides a means to identify where and when myelination deviates from the typical trajectory.

In this work, we have restricted ourselves to single gender data across a restricted age range. This was to ensure a homogeneous sample with which to explore the modeling functions as well as avoid potential gender-based variations in development. It should be noted, however, that while the goal of this work was not to identify potential gender differences in myelination, developmental trajectories have been reported to differ between males and females. For example, Lenroot et al. (2007) observed males and females to have distinct patterns of brain growth, specifically noting males to have an accelerated rate of white matter volume maturation. While volumetric measures, such as white matter volume, reflect broad microstructural composition, the observed gender differences are likely to be influenced by changes in myelin content. Hence, we would expect to find similar gender differences in the trajectories of MWF and a future study investigating the role that gender has on MWF development is of great interest.

Another aspect of the developed model not investigated in this work is generalizability of the model to data acquired from other imaging centers and scanners. Indeed, in order for model-derived MWF maps to have broad applicability and provide meaningful comparisons across data from different groups, values should be consistent across imaging centers and scanners. While the mcDESPOT imaging technique has been reported to have high intra- and inter-site reproducibility (Deoni et al., 2009), none of the data analyzed in this work has been separately acquired at a different imaging facility nor has any participant been imaged on a different MRI scanner. Scanning individuals at different imaging facilities as well as on multiple scanners would not only be of significant interest but necessary to investigate this aspect of the developed model.

In this work, we have outlined a framework that can be used to model myelination trajectories that are derived from MWF measurements in children under the age of 5 years. We have shown that a modified Gompertz growth model provides the most accurate representation of myelin maturation using the BIC parsimony metric and have developed the first 4D atlas of myelin maturation in healthy males under 5 years of age. This work demonstrates the ability to accurately model early myelin maturation trajectories and provides a normative template of typical myelination in healthy male infants across a large age span (between the age of 70 and 1809 days) from which atypical myelin development can be assessed. The resulting model thus provides an important step for understanding ‘typical’ myelin development as well as providing the ability to identify when and where white matter abnormalities occur in neurodevelopmental disorders.

## References

[bb0005] Avants B.B., Epstein C.L., Grossman M., Gee J.C. (2008). Symmetric diffeomorphic image registration with cross-correlation: evaluating automated labeling of elderly and neurodegenerative brain. Med. Image Anal..

[bb0010] Barkovich A.J., Kjos B.O., Jackson D.E., Norman D. (1988). Normal maturation of the neonatal and infant brain: MR imaging at 1.5 T. Radiology.

[bb0015] Barnea-Goraly N., Menon V., Eckert M., Tamm L., Bammer R., Karchemskiy A., Dant C.C., Reiss A.L. (2005). White matter development during childhood and adolescence: a cross-sectional diffusion tensor imaging study. Cereb. Cortex.

[bb0020] Bartzokis G., Lu P.H., Tingus K., Mendez M.F., Richard A., Peters D.G., Oluwadara B., Barrall K.A., Finn J.P., Villablanca P., Thompson P.M., Mintz J. (2010). Lifespan trajectory of myelin integrity and maximum motor speed. Neurobiol. Aging.

[bb0025] Ben Bashat D., Kronfeld-Duenias V., Zachor D.A., Ekstein P.M., Hendler T., Tarrasch R., Even A., Levy Y., Ben Sira L. (2007). Accelerated maturation of white matter in young children with autism: a high b value DWI study. Neuroimage.

[bb0030] Bleasdale J., Nelder J. (1960). Plant population and crop yield. Nature.

[bb0035] Chang L.C., Jones D.K., Pierpaoli C. (2005). RESTORE: robust estimation of tensors by outlier rejection. Magn. Reson. Med..

[bb0040] Chung S., Ying L., Henry R.G. (2006). Comparison of bootstrap approaches for estimation of uncertainties of DTI parameters. Neuroimage.

[bb0045] Courchesne E. (2004). Brain development in autism: early overgrowth followed by premature arrest of growth. Ment. Retard. Dev. Disabil. Res. Rev..

[bb0050] Darki F., Oghabian M.A. (2009). Accurate activation map detection using bootstrap resampling of single fMRI data. Conf. Proc. IEEE Eng. Med. Biol. Soc..

[bb0055] Deoni S.C.L. (2010). Correction of main and transmit magnetic field (B0 and B1) inhomogeneity effects in multicomponent-driven equilibrium single-pulse observation of T1 and T2. Magn. Reson. Med..

[bb0075] Deoni S.C.L., Rutt B.K., Arun T., Pierpaoli C., Jones D.K. (2008). Gleaning multicomponent T1 and T2 information from steady-state imaging data. Magn. Reson. Med..

[bb0080] Deoni S.C.L., Samson R., Wheeler-Kingshot C.A. (2009). Proc 17th Annual Meeting ISMRM, Honolulu.

[bb0070] Deoni S.C.L., Mercure E., Blasi A., Gasston D., Thomson A., Johnson M., Williams S.C.R., Murphy D.G.M. (2011). Mapping infant brain myelination with magnetic resonance imaging. J. Neurosci..

[bb0060] Deoni S.C.L., Dean D.C., O'Muircheartaigh J., Dirks H., Jerskey B.A. (2012). Investigating white matter development in infancy and early childhood using myelin water faction and relaxation time mapping. NeuroImage.

[bb0065] Deoni S.C.L., Matthews L., Kolind S.H. (2013). One component? Two components? Three? The effect of including a nonexchanging "free" water component in multi-component driven equilibrium single pulse observation of T1 and T2. Magn. Reson. Med..

[bb0085] Efron B. (1979). Bootstrap methods: another look at the jackknife. Ann. Stat..

[bb0090] Flechsig P. (1901). Developmental (myelogenetic) localisation of the cerebral cortex in the human subject. Lancet.

[bb0095] Fornari E., Knyazeva M.G., Meuli R., Maeder P. (2007). Myelination shapes functional activity in the developing brain. NeuroImage.

[bb0100] Gareau P.J., Rutt B.K., Karlik S.J., Mitchell J.R. (2000). Magnetization transfer and multicomponent T2 relaxation measurements with histopathologic correlation in an experimental model of MS. J. Magn. Reson. Imaging.

[bb0105] Geng X., Gouttard S., Sharma A., Gu H., Styner M., Lin W., Gerig G., Gilmore J.H. (2012). Quantitative tract-based white matter development from birth to age 2 years. NeuroImage.

[bb0115] Giedd J.N., Rapoport J.L. (2010). Structural MRI of pediatric brain development: what have we learned and where are we going?. Neuron.

[bb0110] Giedd J., Blumenthal J., Jeffries N. (1999). Brain development during childhood and adolescence: a longitudinal MRI study. Nat. Neurosci..

[bb0120] Gompertz B. (1825). On the nature of the function expressive of the law of human mortality, and on a new mode of determining the value of life contingencies. Philos. Trans. R. Soc. Lond..

[bb0125] Haroon H., Morris D., Embleton K. (2009). Using the model-based residual bootstrap to quantify uncertainty in fiber orientations from Q-ball analysis. IEEE Trans. Med. Imaging.

[bb0130] Haroutunian V., Davis K.L. (2007). Introduction to the special section: myelin and oligodendrocyte abnormalities in schizophrenia. Int. J. Neuropsychopharmacol..

[bb0135] Hermoye L., Saint-Martin C., Cosnard G., Lee S.-K., Kim J., Nassogne M.-C., Menten R., Clapuyt P., Donohue P.K., Hua K., Wakana S., Jiang H., van Zijl P.C.M., Mori S. (2006). Pediatric diffusion tensor imaging: normal database and observation of the white matter maturation in early childhood. NeuroImage.

[bb0140] Huang H., Zhang J., Wakana S., Zhang W., Ren T., Richards L.J., Yarowsky P., Donohue P., Graham E., van Zijl P.C.M., Mori S. (2006). White and gray matter development in human fetal, newborn and pediatric brains. NeuroImage.

[bb0145] Hurley S.A., Mossahebi P.M., Samsonov A.A., Alexander A.L., Deoni S.C., Fisher R., Ducan I.D., Field A.S. (2010). Proc. 18th Annual Meeting of the ISMRM. Stockholm, SWE.

[bb0150] Johnson M.H., Munakata Y. (2005). Processes of change in brain and cognitive development. Trends Cogn. Sci. (Regul. Ed.).

[bb0160] Jones D.K. (2008). Tractography gone wild: probabilistic fibre tracking using the wild bootstrap with diffusion tensor MRI. IEEE Trans. Med. Imaging.

[bb0165] Jones D.K., Symms M.R., Cercignani M., Howard R.J. (2005). The effect of filter size on VBM analyses of DT-MRI data. NeuroImage.

[bb0155] Jones D., Knosche T., Turner R. (2013). White matter integrity, fiber count, and other fallocies: the do's and don'ts of diffusion MRI. NeuroImage.

[bb0170] Kirson D., Huk A.C., Cormack L.K. (2008). Quantifying spatial uncertainty of visual area boundaries in neuroimaging data. J. Vis..

[bb0175] Kitzler H.H., Su J., Zeineh M., Harper-Little C., Leung A., Kremenchutzky M., Deoni S.C., Rutt B.K. (2012). Deficient MWF mapping in multiple sclerosis using 3D whole-brain multi-component relaxation MRI. NeuroImage.

[bb0180] Knickmeyer R.C., Gouttard S., Kang C., Evans D., Wilber K., Smith J.K., Hamer R.M., Lin W., Gerig G., Gilmore J.H. (2008). A structural MRI study of human brain development from birth to 2 years. J. Neurosci..

[bb0185] Kolind S., Matthews L., Johansen-Berg H., Leite M.I., Williams S.C.R., Deoni S., Palace J. (2012). Myelin water imaging reflects clinical variability in multiple sclerosis. NeuroImage.

[bb0190] Kolind S., Sharma R., Knight S., Johansen-Berg H., Talbot K., Turner M.R. (2013). http://informahealthcare.com/doi/abs/10.3109/21678421.2013.794843.

[bb0195] Konrad K., Eickhoff S.B. (2010). Is the ADHD brain wired differently? A review on structural and functional connectivity in attention deficit hyperactivity disorder. Hum. Brain Mapp..

[bb0200] Kumar R., Nguyen H., Macey P. (2011). Regional brain axial and radial diffusivity changes during development. J. Neurosci. Res..

[bb0210] Laule C., Leung E., Lis D.K.B., Traboulsee A.L., Paty D.W., MacKay A.L., Moore G.R.W. (2006). Myelin water imaging in multiple sclerosis: quantitative correlations with histopathology. Mult. Scler..

[bb0205] Laule C., Kozlowski P., Leung E., Li D.K.B., MacKay A.L., Moore G.R.W. (2008). Myelin water imaging of multiple sclerosis at 7 T: correlations with histopathology. NeuroImage.

[bb0215] Lebel C., Beaulieu C. (2011). Longitudinal development of human brain wiring continues from childhood into adulthood. J. Neurosci..

[bb0220] Lebel C., Gee M., Camicioli R., Wieler M., Martin W., Beaulieu C. (2012). Diffusion tensor imaging of white matter tract evolution over the lifespan. NeuroImage.

[bb0225] Lenroot R.K., Gogtay N., Greenstein D.K., Wells E.M., Wallace G.L., Clasen L.S., Blumenthal J.D., Lerch J., Zijdenbos A.P., Evans A.C., Thompson P.M., Giedd J.N. (2007). Sexual dimorphism of brain developmental trajectories during childhood and adolescence. NeuroImage.

[bb0230] Levenberg K. (1944). A method for the solution of certain non-linear problems in least squares. Q. Appl. Math..

[bb0235] MacKay A., Laule C., Vavsour I., Bjarnason T., Kolling S., Madler B. (2006). Insights into brain microstructure from the T2 distribution. Magn. Reson. Imaging.

[bb0240] MacKay A.L., Vavasour I.M., Rauscher A., Kolind S.H., Mädler B., Moore G.R.W., Traboulsee A.L., Li D.K.B., Laule C. (2009). MR relaxation in multiple sclerosis. Neuroimaging Clin. N. Am..

[bb0250] Mädler B., Drabycz S., Kolind S., Whittall K. (2008). Is diffusion anisotropy an accurate monitor of myelination? Correlation of multicomponent T2 relaxation and diffusion tensor anisotropy in human brain. Magn. Reson. Med..

[bb0245] Mazziotta J., Toga A., Evans A., Fox P. (2001). A four-dimensional probabilistic atlas of the human brain. J. Am. Med. Inform. Assoc..

[bb0255] Moll N.M., Rietsch A.M., Thomas S., Ransohoff A.J., Lee J.-C., Fox R., Chang A., Ransohoff R.M., Fisher E. (2011). Multiple sclerosis normal-appearing white matter: pathology-imaging correlations. Ann. Neurol..

[bb0260] Odrobina E.E., Lam T.Y.J., Pun T., Midha R., Stanisz G.J. (2005). MR properties of excised neural tissue following experimentally induced demyelination. NMR Biomed..

[bb0265] Paus T., Collins D.L., Evans A.C., Leonard G., Pike B., Zijdenbos A. (2001). Maturation of white matter in the human brain: a review of magnetic resonance studies. Brain Res. Bull..

[bb0270] Richards F. (1959). A flexible growth function for empirical use. J. Exp. Bot..

[bb0275] Sadeghi N., Prastawa M., Fletcher P.T., Wolff J., Gilmore J.H., Gerig G. (2012). Regional characterization of longitudinal DT-MRI to study white matter maturation of the early developing brain. NeuroImage.

[bb0280] Schwarz G. (1978). Estimating the dimension of a model. Ann. Stat..

[bb0285] Smith S. (2002). Fast robust automated brain extraction. Hum. Brain Mapp..

[bb0290] Stannard C., Williams A., Gibbs P. (1985). Temperature/growth relationships for psychrotrophic food-spoilage bacteria. Food Microbiol..

[bb0295] van Buchem M.A., Steens S.C., Vrooman H.A., Zwinderman A.H., McGowan J.C., Rassek M., Engelbrecht V. (2001). Global estimation of myelination in the developing brain on the basis of magnetization transfer imaging: a preliminary study. AJNR Am. J. Neuroradiol..

[bb0300] Vavasour I., Whittall K., Mackay A. (2005). A comparison between magnetization transfer ratios and myelin water percentages in normals and multiple sclerosis patients. Magn. Reson. Med..

[bb0305] Vavasour I.M., Laule C., Li D.K.B., Traboulsee A.L., MacKay A.L. (2011). Is the magnetization transfer ratio a marker for myelin in multiple sclerosis?. J. Magn. Reson. Imaging.

[bb0310] Webb S., Munro C.A., Midha R., Stanisz G.J. (2003). Is multicomponent T2 a good measure of myelin content in peripheral nerve?. Magn. Reson. Med..

[bb0315] Wheeler-Kingshott C.A.M., Cercignani M. (2009). About “axial” and “radial” diffusivities. Magn. Reson. Med..

[bb0320] Whittall K.P., MacKay A.L., Graeb D.A., Nugent R.A., Li D.K., Paty D.W. (1997). In vivo measurement of T2 distributions and water contents in normal human brain. Magn. Reson. Med..

[bb0325] Yakovlev P.I., Lecours A.R., Mankowski A. (1967). Regional Development of the Brain in Early Life.

[bb0330] Yuan Y., Zhu H., Ibrahim J.G., Lin W., Peterson B.S. (2008). A note on the validity of statistical bootstrapping for estimating the uncertainty of tensor parameters in diffusion tensor images. IEEE Trans. Med. Imaging.

[bb0335] Zaaraoui W., Deloire M., Merle M., Girard C., Raffard G., Biran M., Inglese M., Petry K.G., Gonen O., Brochet B., Franconi J.-M., Dousset V. (2008). Monitoring demyelination and remyelination by magnetization transfer imaging in the mouse brain at 9.4 T. MAGMA.

[bb0340] Zhang J., Kolind S., Mackay A.L. (2013). Proc. 21st Annual Meeting of the ISMRM. Salt Lake City, UT, USA.

[bb0345] Zilles K., Amunts K. (2013). Individual variability is not noise. Trends Cogn. Sci..

